# Unconscious neural processing differs with method used to render stimuli invisible

**DOI:** 10.3389/fpsyg.2014.00601

**Published:** 2014-06-16

**Authors:** Sergey V. Fogelson, Peter J. Kohler, Kevin J. Miller, Richard Granger, Peter U. Tse

**Affiliations:** ^1^Department of Psychological and Brain Sciences, Dartmouth CollegeHanover, NH, USA; ^2^Department of Psychology, Stanford UniversityStanford, CA, USA; ^3^Princeton Neuroscience Institute, Princeton UniversityPrinceton, NJ, USA

**Keywords:** continuous flash-induced suppression, flicker fusion, fMRI, categorical representation, multi-voxel pattern analysis, consciousness

## Abstract

Visual stimuli can be kept from awareness using various methods. The extent of processing that a given stimulus receives in the absence of awareness is typically used to make claims about the role of consciousness more generally. The neural processing elicited by a stimulus, however, may also depend on the method used to keep it from awareness, and not only on whether the stimulus reaches awareness. Here we report that the method used to render an image invisible has a dramatic effect on how category information about the unseen stimulus is encoded across the human brain. We collected fMRI data while subjects viewed images of faces and tools, that were rendered invisible using either continuous flash suppression (CFS) or chromatic flicker fusion (CFF). In a third condition, we presented the same images under normal fully visible viewing conditions. We found that category information about visible images could be extracted from patterns of fMRI responses throughout areas of neocortex known to be involved in face or tool processing. However, category information about stimuli kept from awareness using CFS could be recovered exclusively within occipital cortex, whereas information about stimuli kept from awareness using CFF was also decodable within temporal and frontal regions. We conclude that unconsciously presented objects are processed differently depending on how they are rendered subjectively invisible. Caution should therefore be used in making generalizations on the basis of any one method about the neural basis of consciousness or the extent of information processing without consciousness.

## Introduction

Blindsight (Weiskrantz, 1990) reveals that some patients who lack conscious vision can have residual unconscious processing. A similar state of affairs can be achieved with normal subjects when stimuli are presented in ways that restrict objects from reaching awareness. Since at least the time of the earliest studies on subliminal perception (Peirce and Jastrow, 1884), researchers have investigated the extent of neural processing that occurs when stimuli are rendered invisible to an observer. This work has stimulated the discovery of multiple ways of eliminating a stimulus from awareness, each with its own specific methodological advantages and disadvantages.

Forward and backward masking allow a stimulus to be rendered subjectively invisible by briefly presenting other highly salient stimuli just before or after the to-be-masked stimulus. Although this method is very effective at rendering a stimulus invisible, the stimulus of interest can be presented for only very brief (<100 ms) durations (Macknik, 2006). Binocular rivalry allows for longer durations of stimulus invisibility by presenting a different stimulus to each eye. Typically, only one eye's input is seen at a time, rendering input to the other eye invisible (Blake, 2001). Although this method extends the duration of stimulus invisibility, stimulus visibility varies stochastically, making both its onset and duration unpredictable (Blake et al., 1971).

A recent elaboration of the binocular rivalry technique, called “continuous flash suppression” (CFS) (Fang and He, 2005; Tsuchiya and Koch, 2005; Koch and Tsuchiya, 2007), presents a temporally dynamic high-contrast image sequence to one eye, while the stimulus of interest is presented to the other eye. This largely removes the unpredictability of standard binocular rivalry between static images, because the dynamic stimulus is so salient that it completely dominates perception in most cases; subjects rarely report seeing the other stimulus at all. This also means that the duration of invisibility can be extended substantially, sometimes for as long as several minutes (Tsuchiya and Koch, 2005). Both binocular rivalry and CFS, however, rely on presenting a stimulus of non-interest to one eye, which might not always be desirable, especially with CFS where this stimulus needs to be highly salient.

This can be avoided by using dichoptic color masking, which involves showing isoluminant, oppositely colored versions of the *same* stimulus to each eye (Moutoussis and Zeki, 2002; Schurger et al., 2010). The disadvantage of this method is that images must be low-contrast and low-spatial frequency to effectively render the stimulus of interest invisible (Schurger et al., 2010). In a modification of this technique (Hoshiyama et al., 2006), which we will refer to as “chromatic flicker fusion” (CFF), two isoluminant, oppositely colored stimuli are simultaneously presented to both eyes, one image at a time, at a temporal frequency above the flicker fusion threshold (~30 Hz). The two images will fuse together such that the image is perceived to be uniformly colored and unchanging (Hecht and Verrijp, 1933). This allows stimuli of higher contrast and higher spatial frequency to be displayed, with the additional advantage that the stimulus of interest is displayed continuously to both eyes.

Although each of the aforementioned methods effectively renders a stimulus invisible to the observer, this is accomplished in profoundly different ways. To the best of our knowledge, the possibility that different methods of rendering a stimulus invisible can lead to differential residual neural processing in the absence of awareness has only been addressed to a limited extent, and only in rhesus macaques, in the literature (Macknik and Livingstone, 1998), and work in humans has only addressed behavioral differences between distinct stimulus invisibility-inducing paradigms (Faivre et al., 2012). Here we use two of these methods, CFS and CFF, to render identical stimuli from two distinct categories (faces and tools) invisible in human beings, and address the possibility of their differential neural processing. Both methods have been shown to allow residual neural processing of stimuli that are invisible to the observer, and are equally effective at rendering relatively high contrast and high spatial frequency stimuli invisible for several seconds or longer (Fang and He, 2005; Jiang et al., 2007; Sterzer et al., 2008).

Multivariate pattern analysis (MVPA) can be used to evaluate the extent to which stimulus information can be recovered from functional neuroimaging data (Cox and Savoy, 2003). We used MVPA to determine whether stimulus category information (in our case, faces or tools) was present in anatomical regions throughout the brain across our three presentation methods (visible, invisible during CFF, and invisible during CFS). If stimulus category can be recovered in a brain region under one presentation condition, but not another, this would suggest that the two conditions lead to differential neural processing in that region. Thus, identifying regions that contain stimulus information under one condition, but not another, allows for the discovery of potential differences in the unconscious processing elicited by different presentation methods.

We were also able to look within areas where classification was in fact possible with more than one presentation method and ask the additional question of whether there were commonalities in the representations of category information elicited by visible and both invisible presentation methods. To accomplish this, we used a cross-method classification approach and tested whether a pattern classifier trained on data from one presentation method could successfully predict stimulus category in a test dataset from the other presentation method. Successful cross-method classification may indicate that different presentation methods yield dissociable representations within the same brain region.

Several experimental outcomes are possible. It could be that stimuli are processed in the same way, regardless of how they are rendered invisible. This would be the case if a single, presumably cortical, bottleneck for conscious visual processing exists, beyond which there is no processing of unconscious stimuli. In this case we would expect category information to be present in largely the same subset of brain areas regardless of the method used to render the stimuli invisible. On the other hand, if unconscious processing depends on how the stimuli are rendered invisible, we would expect to see areas where one method allows classification, but the other does not, and other areas where the opposite is the case. Such a result would suggest that properties of the method used to induce stimulus invisibility had a significant effect on the propagation of stimulus information through cortex.

We collected full-brain fMRI data while presenting subjects with pictures of faces and tools under three conditions (visible, invisible during CFS, invisible during CFF). We found that the cortical and subcortical areas that distinguish between unconsciously processed face and tool categories were largely nonoverlapping between the CFS and CFF conditions. Although visible stimulus category information was present throughout cortex, stimulus information rendered invisible via CFS was recoverable exclusively from occipital cortex, whereas stimulus information rendered invisible via CFF was recoverable from occipital, temporal, and frontal regions. These results suggest that invisible objects are processed differently depending on the method used to render them invisible.

## Materials and methods

### Participants

Seventeen healthy subjects (9 men) aged between 19 and 29 (mean age 24.3 years) participated in the experiment. All subjects had normal or corrected-to-normal vision and gave written, informed consent in accordance with procedures and protocols for testing human subjects, approved by the Institutional Review Board of Dartmouth College.

### Experimental design

Stimuli were human faces (2 male, 2 female) and common tools (spoon, fork, hammer, wrench) drawn as either red or green outlines on a yellow-green background. Faces subtended approximately 4.5° by 8° of visual angle, whereas tool stimuli were ~2.5° by ~8° and were always elongated along the vertical axis (Sakuraba et al., 2012). All images were presented within an 8° by 8° viewing window. Additionally, each image contained a central white fixation cross that subtended 0.75° by 0.75° of visual angle. Stimuli were always presented for 2 s during a given trial, regardless of trial type.

**Figure 1:**
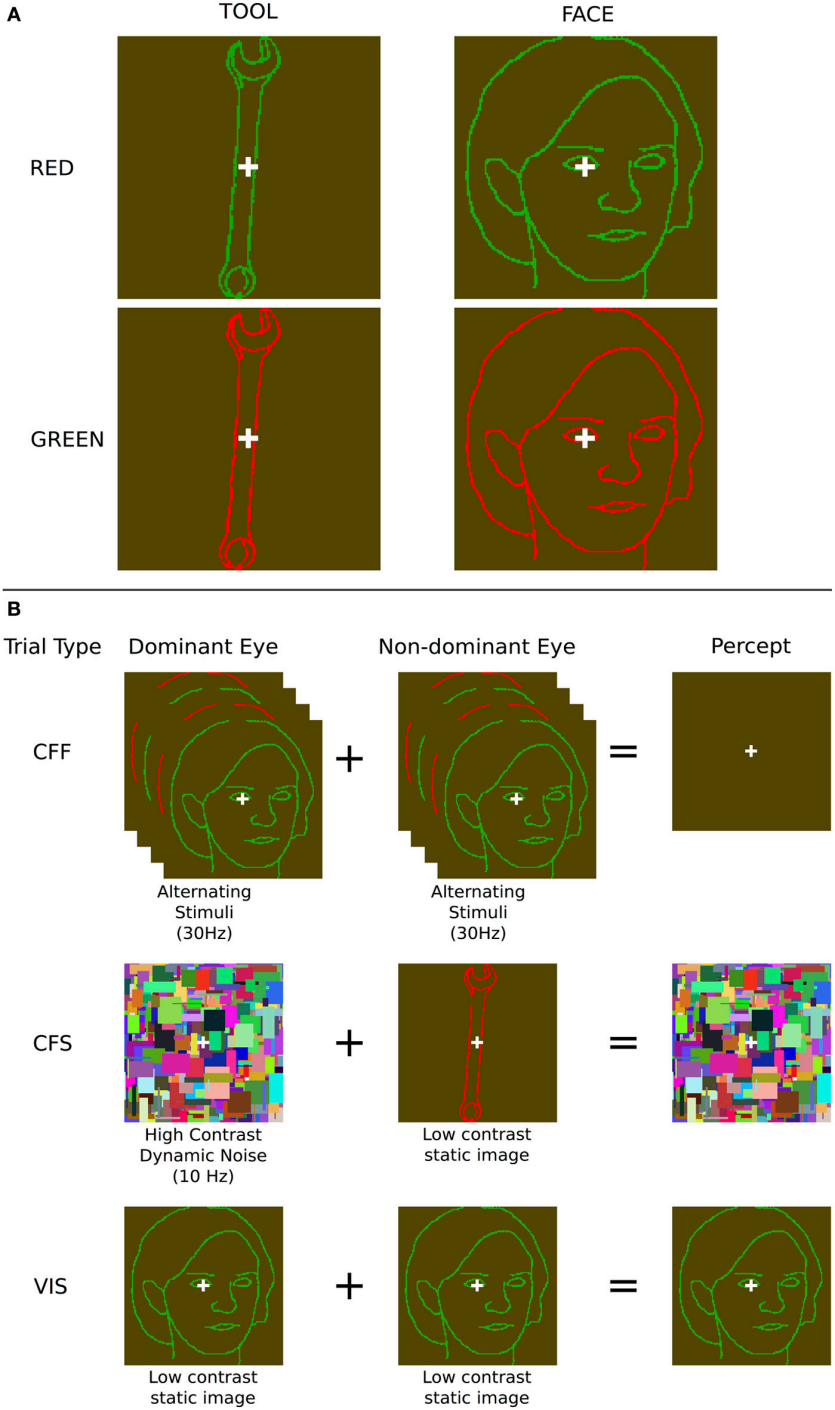
**Stimuli and presentation methods used. (A)** Example face and tool stimuli—note that the contrast of the outline stimuli, which were in fact perceptually isoluminant, is enhanced here for illustration purposes. **(B)** The three presentation methods: During chromatic flicker fusion trials, isoluminant images were flickered in both eyes continuously at 30 Hz and rendered invisible; During continuous flash suppression trials, an object image was presented to the non-dominant eye and a high contrast pattern changing at 10 Hz was presented to the dominant eye, rendering the object image invisible; During visible trials, identical object images were presented to both eyes. During all trials, image visibility was tested using objective behavioral measurements.

We used two methods to render face and tool stimuli invisible to subjects. During CFF, subjects were presented with two isoluminant oppositely colored (red and green) images that flickered dynamically in counter-phase with each other at 30 Hz. Since the flicker rate is above the critical flicker fusion threshold for color (Jiang et al., 2007), this manipulation led subjects to perceive a continuous uniform dark-green colored field, with a white fixation cross in the center (Figure 1). Previous results show that this method is effective at rendering stimuli invisible, while still allowing unconscious processing (Moutoussis and Zeki, 2002; Jiang et al., 2007). In order to ensure that the values of green and red used for the CFF stimuli were isoluminant so that they could support fusion, they were adjusted prior to the experiment to be perceptually isoluminant for each subject individually. This isoluminance task was performed using heterochromatic flicker photometry with alternating red and green squares (Ives, 1911; Lee et al., 1988). During this calibration task, subjects saw a single square that rapidly flickered (20 Hz) between red and green; they then had to adjust the luminance of the green color until the magnitude of flickering between the two colors was perceptually minimized. At the minimal flicker point, subjects perceived a uniform, minimally flickering square with a color approximately halfway between the two original colors (a dark greenish yellow color). Once this value was determined, all stimuli used for each subject across all presentation methods had identical contrast values with the specific luminance values found during calibration.

We contrasted this method with CFS, which has regularly been shown to render a large variety of stimulus types invisible to the observer (Koch and Tsuchiya, 2007), evident in both behavioral (Tsuchiya and Koch, 2005; Almeida et al., 2008; Bahrami et al., 2010) and neuroimaging data (Fang and He, 2005; Jiang and He, 2006; Sterzer et al., 2008). We presented a high-contrast dynamically changing (10 Hz) “Mondrian” pattern, consisting of randomly positioned rectangles of various sizes and colors, in each subject's dominant eye, and showed isoluminant red/green face or tool stimuli identical to those used for the CFF condition to the non-dominant eye. This rendered the stimulus presented to the non-dominant eye invisible (Figure 1). A white fixation cross, identical to that used in the CFF condition, was placed on top of each Mondrian stimulus. Both of these manipulations rendered faces and tools invisible and allowed us to use fMRI to investigate cortical and subcortical object processing in the absence of awareness. For comparison, we also measured patterns of activity when subjects were presented with only one of the two CFF stimuli (red or green), without any flicker, which rendered the stimuli fully visible.

### Data acquisition

Structural and functional data were collected using a Philips 3T Achieva scanner with a 32-channel head coil. BOLD signals were measured with an EPI (echo-planar imaging) sequence [35 axially oriented slices, 3.0 × 3.0 mm in-plane voxel resolution, 3.5 mm slice thickness, no gap, interleaved slice acquisition, FOV = 240 × 240 × 122, TE = 35 ms, flip angle = 90°, TR = 2000 ms, acquired matrix size = 80 × 80, reconstructed matrix size = 80 × 80, P reduction (RL) sense factor of 2, S reduction (FH) sense factor of 1] and structural anatomical scans were acquired using an MPRAGE sequence (220 axial slices, 0.94 × 0.94 mm in-plane voxel resolution, 1 mm slice thickness, acquired matrix size = 240 × 187, reconstructed matrix size = 256 × 256, FOV = 240 × 188 × 220, TR/TE = 8.2/3.7). Inside the scanner bore, subjects viewed stimuli through MR-compatible VisuaStimDigital binocular presentation goggles (Resonance Technology Inc., Los Angeles, CA). We used a slow event related design where each 2 s trial was followed by a 6 or 8 s blank ISI with a fixation cross (both ISIs had an equal probability of occurring and one was randomly chosen following each trial). All 8 stimuli were shown twice per run using each experimental method (fully visible, CFF, CFS). For the visible and CFS conditions one of the two presentations of each stimuli utilized the green outline CFF stimulus, and the other presentation utilized the red outline CFF stimulus, while during the CFF condition both presentations of each were identical (red and green outline flickering). This gave a total of 48 stimuli per run (8 stimuli × 2 different color outlines × 3 presentation methods). Trials for CFS, CFF, and visible conditions occurred in random order throughout each experimental run. A near-optimal run order was generated by creating millions of possible run orders and evaluating them according to first and second-order correlations in the stimulus order at the category level (so that the probability that a face followed a face or a tool followed a tool was minimized) and selecting the order with the minimum first and second-order correlation (for a first-order correlation, this makes the probability that a face followed a tool roughly the same as the probability that it would follow a face; for a second-order correlation, this made it so that the probability of a face followed by either a face or a tool and again followed by a face is roughly the same as the probability that the second succeeding stimulus is a tool). Stimulus orders were generated on a per-subject basis, such that each subject saw a different first-order and second-order correlation-minimized ordering of images. Following each presentation, subjects indicated stimulus category with one button box, guessing in the cases where they had no awareness of the stimulus. Using a second button box, subjects were also asked to indicate whether stimulus breakthrough occurred (that is, whether they saw anything other than a Mondrian pattern or a uniformly colored field). During visible trials, subjects were required to use this second button box to indicate that a stimulus was clearly visible.

Experimental runs lasted for 242 2-s TRs (484 s). All subjects underwent 8 experimental runs acquired in a single scanning session. Any CFS or CFF trials during which subjects indicated that breakthrough had occurred were eliminated from further analysis. To further ensure that stimuli were indeed completely invisible to subjects during unconscious trials, any experimental runs for which subjects were able to guess the category of invisible stimuli with greater than 75% accuracy (cumulative binomial *p* < 0.05) were not used in further analyses for that trial type. The 75% criterion was chosen to exclude runs where button presses during unconscious trials could be used to decode stimulus category with greater than chance accuracy. This occurred quite rarely (three runs for three different subjects, out of a total of 136 runs of data gathered across all subjects). All fully visible trials were included, regardless of subject response.

### Data preprocessing

Functional imaging data were preprocessed using FSL (Jenkinson et al., 2011). Preprocessing included motion correction, high pass filtering (>0.01 Hz) and spatial smoothing with a 5 mm Gaussian kernel. After preprocessing, multi-voxel pattern analysis was performed on the data with a linear support vector machine (SVM) pattern classifier as implemented in PyMVPA (Hanke et al., 2009) using all default parameters (see Within-method and cross-method category classification). Data from the second and third TR (4 and 6 s) following stimulus onset were used for classification. All trials within a run were averaged to create a single pattern of activity per category per run.

All individual ROI masks were generated using FreeSurfer's automatic anatomical parcellations of both cortical gray-matter and subcortical regions (Destrieux et al., 2010), allowing us to perform the analyses within each subject's own anatomical space and to avoid the loss of statistical power and normalization inaccuracies associated with group-level analyses done in a standard space. For a given anatomical region, masks from both hemispheres were combined to yield a single bilateral ROI. This yielded a total of 67 bilateral ROIs per subject. A cortical surface reconstruction of a standard anatomical template (the MNI 152-brain average atlas) was also created using SUMA (Saad and Reynolds, 2012) for illustrating group-level ROI results.

### Within-method and cross-method category classification

All analyses were performed in each subject's native space using the bilateral anatomically defined regions of interest (ROI) described above. All pattern classifications where performed using a linear SVM classifier, as described above. First, classification of faces and tools was performed using only the data acquired during the visible presentation method. This was done in order to identify the set of areas that contained visible categorical information, and to limit the number of comparisons performed when analyzing the conditions where there was no awareness (that is, during either CFF or CFS trials). The assumption was that areas where categorical information was present in the visible case would make the best candidates for investigating the presence of information in the absence of visual awareness. All classifications were performed using leave-one-run-out cross-validation and the resulting d-prime values across all subjects were compared with chance accuracy using a one-sided *t*-test. The resulting *p*-values (during the visible case) were then corrected for multiple comparisons using False Discovery Rate (FDR) to get corrected *p*-values. Those ROIs that survived this initial correction (corrected *p* < 0.05) were then explored in an exactly analogous manner using the data acquired during the two invisible presentation methods (CFF and CFS). Furthermore, paired *t*-tests comparing each display method were also performed in those cases where significant classification occurred. In order to ensure that actual categorical information was being captured for all three conditions, we did the classification such that there was no overlap at the individual-exemplar level between the trials used for the training and test data sets given to the classifier. Specifically, we split each run so that we trained the classifier on one specific set of exemplars across all but one run, and then tested on the left-out exemplars for the held-out run. This same method was repeated on the other set of exemplars (that which was not used for training), with testing happening on the remaining (not trained exemplars) with the same kind of run splits (all but one run training, held out run for testing). This was done for every possible combination of training and testing runs. The two kinds of cross-validation were then averaged together to come up with a single averaged cross-validated cross-exemplar d-prime value. If there were not at least two usable exemplars within a run for a given presentation method, that run was thrown out from analysis for that presentation method (this was only done for 4 functional runs across all 18 subjects). Splitting the data in this way forced the classifier to use voxel patterns that were at least somewhat removed from the most simple low-level identifying features of the stimuli.

In addition, three different kinds of cross-method classification analyses were performed: (1) between invisible presentation methods, (2) invisible to visible, and (3) visible to invisible. As was the case with the within-method classification, cross-method classification was limited to those ROIs that were significant (after FDR-correction) in the visible case. Again, results were FDR-corrected across ROIs and within each analysis. The “between invisible” classification tested whether any regions were capable of distinguishing invisible faces from invisible tools when a classifier was trained on one of the methods (CFF or CFS) and tested on a held-out run that included only trials from the other method (CFS or CFF, respectively). In the “invisible to visible” classification, we trained the classifier on trials from one of the invisible methods (CFF or CFS) and attempted to classify the category (face or tool) during a visible presentation within a given ROI. In “visible to invisible” classification, the approach was exactly the same, except the training and testing patterns were swapped (training was performed on visible trials and testing was performed on one of the invisible methods).

## Results

### Behavioral results

Behavioral data collected during scanning show that subjects were at chance when guessing stimulus category during both invisible conditions (mean accuracy across all trials and subjects for CFS: 49.7%, *p* = 0.9; and CFF: 49.4%, *p* = 0.29, one-sided *t*-test against chance accuracy) and at ceiling (99.4%, *p* < 0.0001, one-sided *t*-test against chance accuracy) during the visible condition. Subjects rarely reported seeing the faces or tools during either invisible condition (3.1% of trials for CFS and 4.3% of trials for CFF across all subjects) and almost always saw stimuli presented during the visible condition (96.2% of trials). We conclude that both methods successfully rendered the stimuli completely invisible.

### Category classification within each presentation method

**Figure 2:**
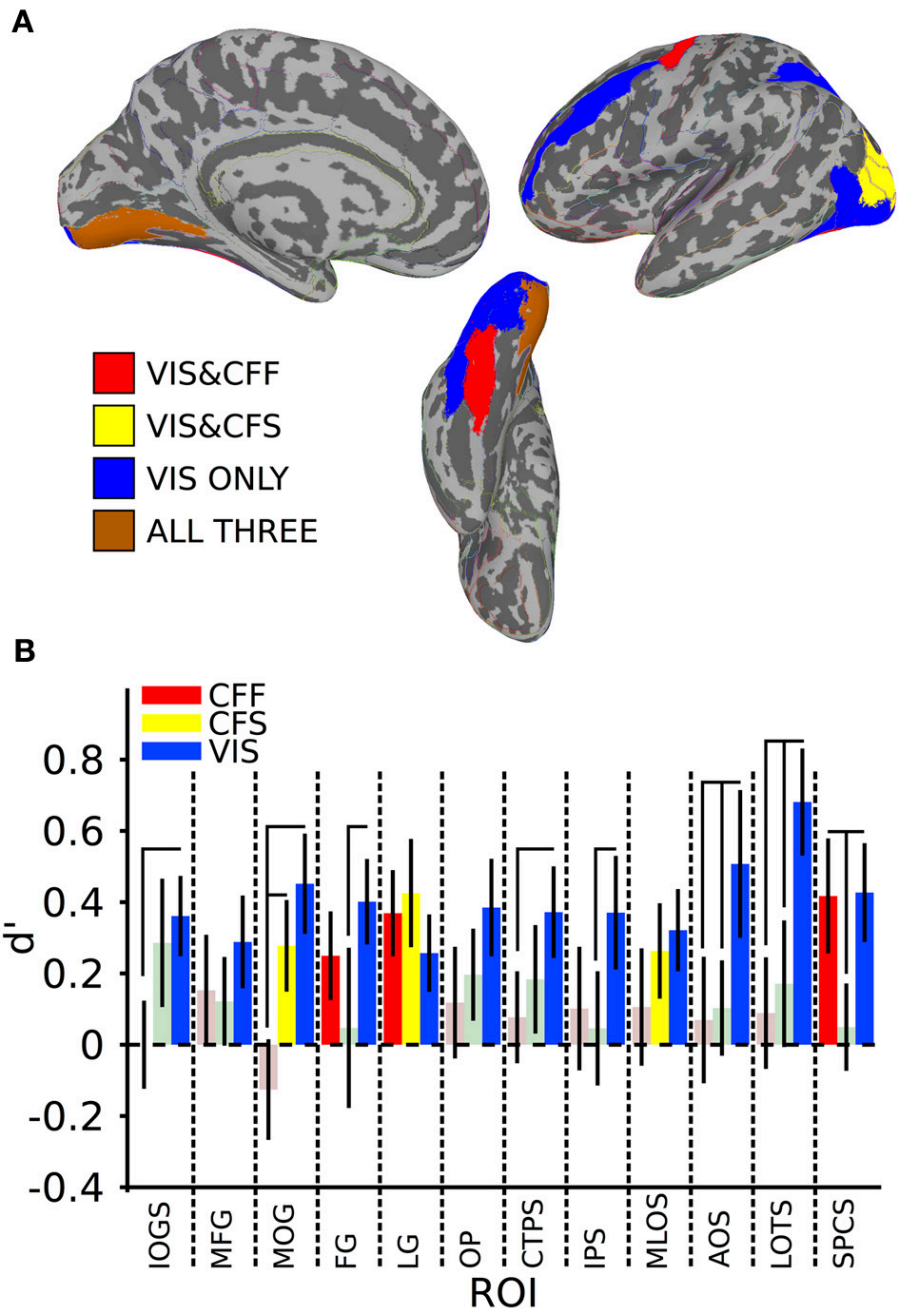
**Within-method classification results. (A)** Colored anatomical ROIs indicate regions where classification of stimulus category was significantly above-change (*p* < 0.05 FDR-corrected) for each presentation method. **(B)** Bar graphs showing the classification accuracy for each method within all ROIs where above-chance classification was possible for the visible presentation method. Saturated (yellow, blue, and red) colors indicate significant classification, whereas desaturated (light red, light green) colors indicate non-significant classification. Full region names for the ROI abbreviations can be found in Table 1.

In order to identify areas across the entire brain that distinguished faces from tools during the visible case, we used an exhaustive region-of-interest (ROI) approach. Linear SVM classification was performed within 67 anatomically defined bilateral ROIs (see Methods). All reported classification results have been FDR-corrected for multiple comparisons (*p* < 0.05) as described in the methods. Our analysis revealed a subset of occipital, temporal, parietal, and frontal ROIs where multivariate pattern classification could distinguish faces from tools when the stimuli were clearly visible. In occipital cortex, these ROIs included the inferior occipital gyrus and sulcus [*t*_(16)_ = 3.21 *p* < 0.05], middle occipital gyrus [*t*_(16)_ = 3.21, *p* < 0.05], lingual gyrus [*t*_(16)_ = 2.37, *p* < 0.05], occipital pole [*t*_(16)_ = 2.81, *p* < 0.05], middle and lunate sulci [*t*_(16)_ = 2.79, *p* < 0.05], and the anterior occipital sulcus [*t*_(16)_ = 2.45, *p* < 0.05] (see Figure 2). In temporal cortex, these included the fusiform gyrus [*t*_(16)_ = 3.35, *p* < 0.05], posterior transverse collateral sulcus [*t*_(16)_ = 2.89, *p* < 0.05], and the lateral occipitotemporal sulcus [*t*_(16)_ = 4.54, *p* < 0.05]. Three additional ROIs outside of occipitotemporal cortex were also identified as containing patterns that could reliably distinguish visible faces from tools: the intraparietal sulcus [*t*_(16)_ = 2.33, *p* < 0.05], the superior part of the precentral gyrus [*t*_(16)_ = 3.08, *p* < 0.05], and the middle frontal gyrus [*t*_(16)_ = 2.22, *p* < 0.05] (see Figure 2).

**Table 1:** ROI names, *t*-values, and mean accuracies for all significant within-method classifications, ^*^Indicates FDR-corrected *p* < 0.05.

	**Full ROI name**	**CFF mean**	**CFF *t*-value**	**CFS mean**	**CFS *t*-value**	**VIS mean**	**VIS *t*-value**	**Size cm^∧^2**
IOGS	Inferior occipital gyrus and sulcus	0	0	0.29	1.59	0.36	3.21^*^	23.96
MFG	Middle frontal gyrus	0.15	0.98	0.12	0.98	0.29	2.21^*^	64.96
MOG	Middle occipital gyrus	−0.13	−0.89	0.28	2.16^*^	0.45	3.21^*^	33.69
FG	Fusiform gyrus	0.25	2.00^*^	0.05	0.21	0.40	3.35^*^	27.08
LG	Lingual gyrus	0.37	3.05^*^	0.43	2.80^*^	0.26	2.37^*^	42.04
OP	Occipital pole	0.12	0.75	0.2	1.52	0.39	2.81^*^	38.05
CTPS	Posterior transverse collateral sulcus	0.08	0.60	0.18	1.21	0.37	2.90^*^	8.36
IPS	Intraparietal sulcus	0.1	0.58	0.05	0.29	0.37	2.33^*^	55.58
MLOS	Middle occipital and lunate sulci	0.11	0.64	0.26	1.97^*^	0.32	2.79^*^	17.84
AOS	Anterior occipital sulcus	0.07	0.39	0.1	0.77	0.51	2.45^*^	13.24
LOTS	Lateral occipitotemporal sulcus	0.08	0.57	0.18	0.96	0.68	4.54^*^	17.66
SPCS	Precentral sulcus, superior part	0.42	2.58^*^	0.05	0.40	0.43	3.08^*^	24.32

**Figure 3:**
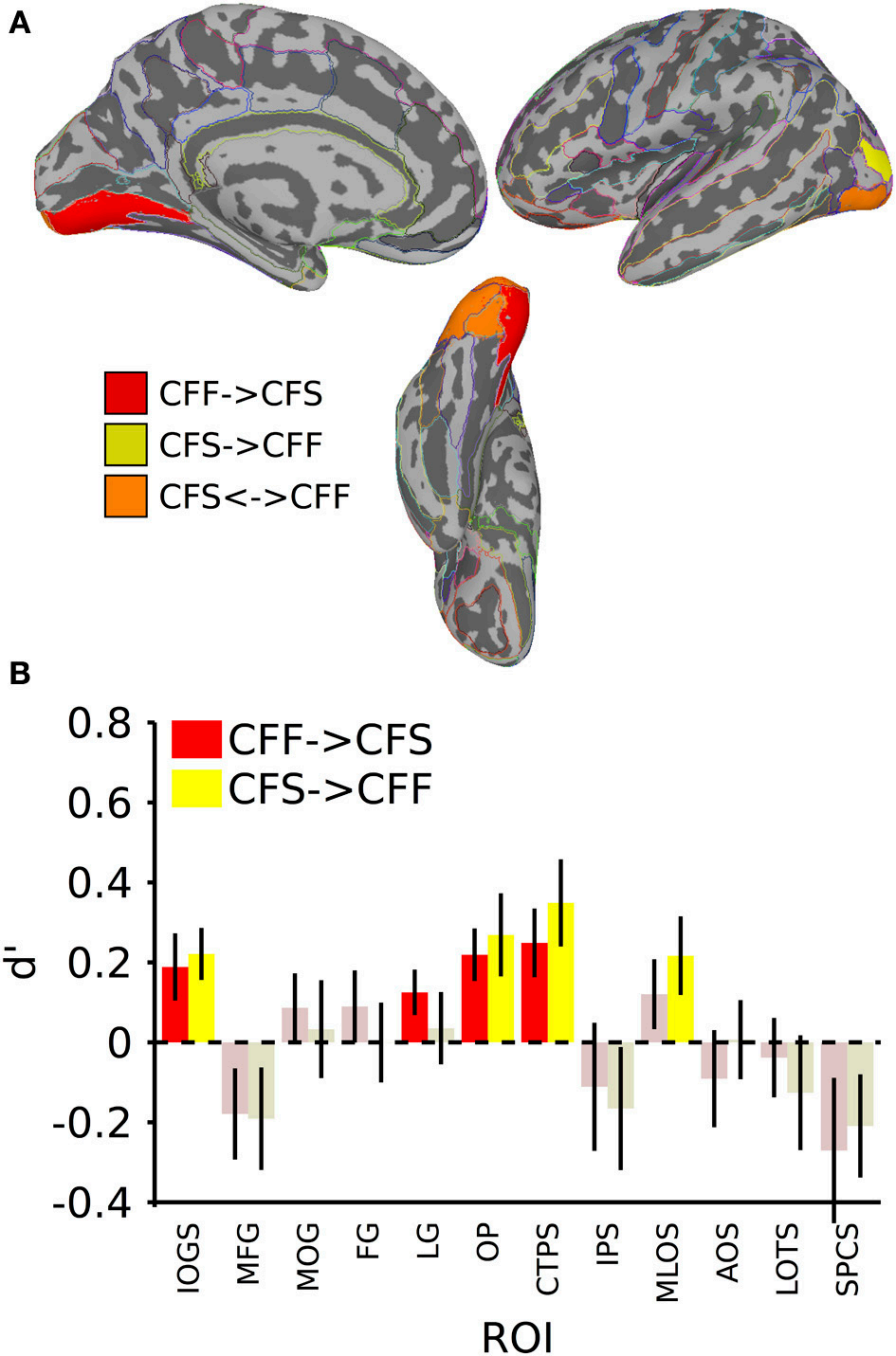
**Results for classification across invisible presentation methods. (A)** Colored anatomical ROIs indicate regions where category classification was significantly above-chance (*p* < 0.05 FDR-corrected) when performing cross-method category classification between the invisible methods (CFF and CFS). **(B)** Bar graphs of all regions where significant cross-method category classification was possible. Full region names for the ROI abbreviations can be found in Table 2. Saturated (yellow and red) colors indicate significant classification, whereas desaturated (light red, light green) colors indicate non-significant classification.

**Figure 4:**
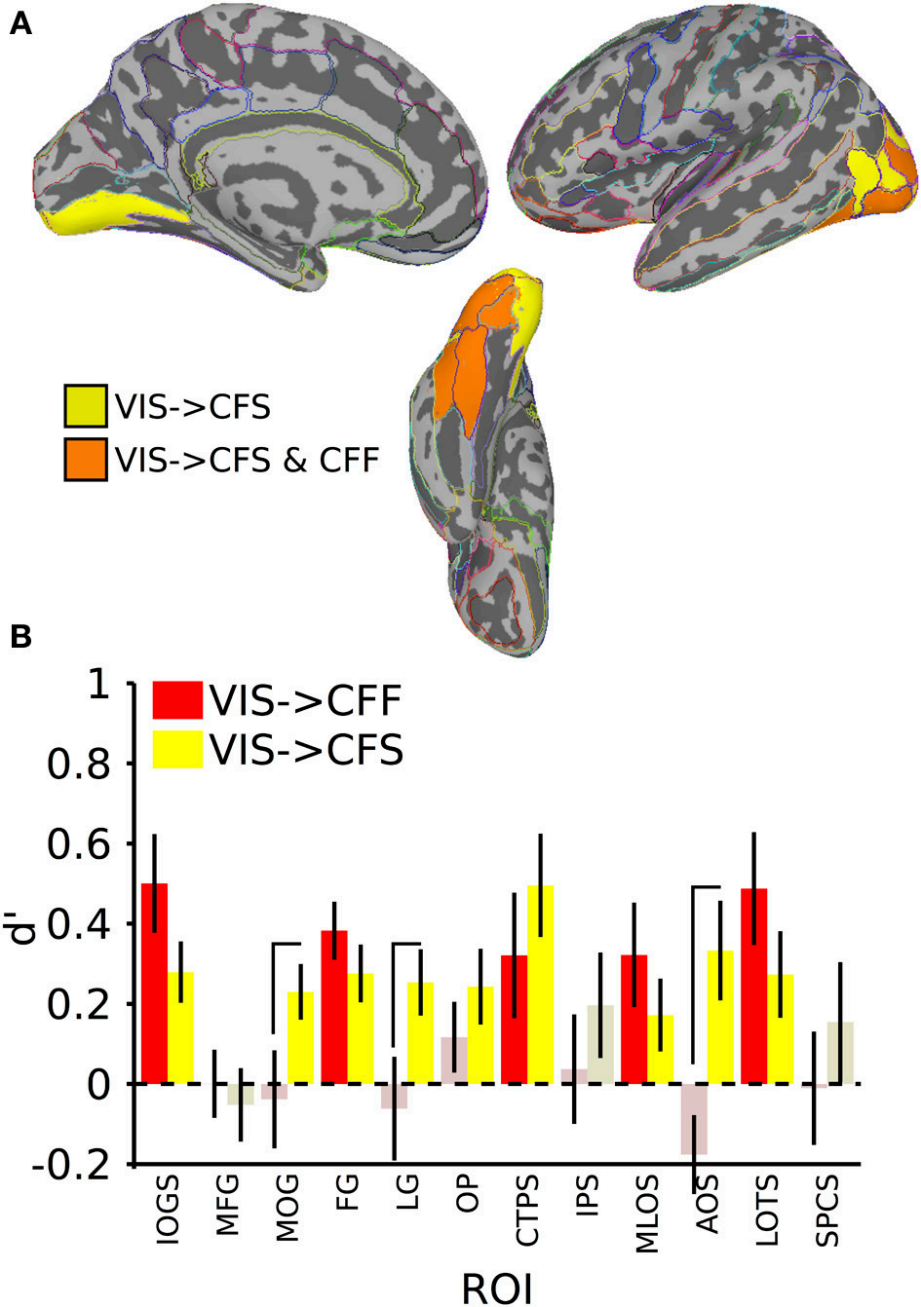
**Results for classification from visible to invisible presentation methods. (A)** Colored anatomical ROIs indicate regions where category classification was significantly above-chance (*p* < 0.05 FDR-corrected) when performing cross-method category classification from visible to either invisible presentation methods. **(B)** Bar graphs of all regions where significant cross-method category classification was possible. Full region names for the ROI abbreviations are found in Table 2. Saturated (yellow and red) colors indicate significant classification, whereas desaturated (light red, light green) colors indicate non-significant classification.

*The first column is the abbreviation used for the given area in Figure 2. Areas are arranged in the same order as that found in the bar graphs in Figures 2–4*.

A subset of these regions also showed significant category classification in the absence of stimulus awareness. Categorical classification was possible when subjects were shown CFS-masked images in the middle occipital gyrus [*t*_(16)_ = 2.16, *p* < 0.05], the middle occipital and lunate sulci [*t*_(16)_ = 2.00, *p* < 0.05], and in the lingual gyrus [*t*_(16)_ = 2.80, *p* < 0.05]. Categorical classification was also possible when subjects viewed CFF-masked images in the lingual gyrus [*t*_(16)_ = 2.01, *p* < 0.05], as well as in the fusiform gyrus [*t*_(16)_ = 3.05, *p* < 0.05] and in the superior part of the precentral sulcus [*t*_(16)_ = 2.58, *p* < 0.05] (see Figure 2).

In several areas, categorical decoding was significantly more robust when objects were completely visible rather than when they were made consciously invisible using either of the other methods. Visible objects were significantly more decodable than objects presented during CFF in the inferior occipital gyrus [*t*_(16)_ = 2.08, *p* < 0.05], middle occipital gyrus [*t*_(16)_ = 3.05, *p* < 0.05], posterior transverse collateral sulcus [*t*_(16)_ = 2.08, *p* < 0.05], and the lateral occipitotemporal sulcus [*t*_(16)_ = 2.48, *p* < 0.05]. In a different subset of regions, visible objects were significantly more decodable than objects presented during CFS, specifically, the fusiform gyrus [*t*_(16)_ = 1.80, *p* < 0.05], the intraparietal sulcus [*t*_(16)_ = 1.82, *p* < 0.05], in the lateral occipitotemporal sulcus [*t*_(16)_ = 2.15, *p* < 0.05], and in the superior part of the precentral sulcus [*t*_(16)_ = 2.22, *p* < 0.05].

Finally, in several regions, the two methods for rendering stimuli invisible were significantly distinguishable from each other. Thus, CFF-based trial categorical decoding was significantly more robust than CFS-trial categorical decoding in the superior part of the precentral sulcus [*t*_(16)_ = 1.99, *p* < 0.05]. On the other hand, CFS-trial categorical decoding was significantly more robust than CFF-trial categorical decoding in the middle occipital gyrus.

To summarize, we found a set of regions within the occipital, temporal, parietal, and frontal lobes that could distinguish fMRI voxel patterns of activity for faces from tools when those objects were clearly visible. Within this set of regions, only a single occipital region, the lingual gyrus, showed the presence of categorical information across all three presentation methods: the visible viewing condition and both invisible conditions in which subjects were completely prevented from consciously identifying the category of the presented objects. Furthermore, visible category information was significantly stronger than category information found during either invisible presentation method in several regions, depending on the method: inferior occipital gyrus, middle occipital gyrus, and posterior transverse collateral sulcus for CFF trials, and in the superior part of the precentral sulcus during CFS trials. In the lateral occipitotemporal sulcus, visible category information was significantly stronger than during both invisible presentation methods. Distinct subsets of regions permitted classification of category information in the two invisible cases. When stimuli were rendered invisible using CFF, categorical information was extractable from the fusiform gyrus and from the superior part of the precentral sulcus, however, only in the latter case was the presence of the categorical information significantly more robust than in the CFS case. When CFS was used, categorical information was present exclusively in occipital regions on the lateral occipital surface (middle occipital gyrus and middle occipital and lunate sulci), but was only significantly more robust than during CFF trials in the middle occipital gyrus.

### Cross-method category classification

We also tested whether patterns of activity for faces and tools could be reliably recovered across presentation methods. We found that a subset of the ROIs described in the previous section, were capable of reliably recovering category information across the invisible presentation methods. Several ROIs showed the presence of information that could reliably distinguish the categories when cross-presentation training and testing regimes were performed in either direction [occipital pole: CFS->CFF: *t*_(16)_ = 2.59, *p* < 0.05; CFF->CFS: *t*_(16)_ = 3.33, *p* < 0.05; inferior occipital gyrus and sulcus: CFS->CFF: *t*_(16)_ = 3.39, *p* < 0.05; CFF->CFS: *t*_(16)_ = 2.25, *p* < 0.05; posterior transverse collateral sulcus: CFS->CFF: *t*_(16)_ = 3.20, *p* < 0.05; CFF->CFS: *t*_(16)_ = 2.90, *p* < 0.05]. In two separate occipital ROIs, categories could only be distinguished either when training on CFF and testing on CFS trials [lingual gyrus—*t*_(16)_ = 2.19, *p* < 0.05] or when training on CFS and testing on CFF trials [middle occipital and lunate sulci—*t*_(16)_ = 2.21, *p* < 0.05]. These data are summarized in Figure 3; *t*-values and mean accuracies for all significant comparisons can be found in Table 2.

**Table 2:** **Names and t-values for all ROIs where at least one of the between-method classifications was significant**.

	**Full ROI name**	**CFS->CFF mean d′**	**CFS->CFF *t*-value**	**CFF->CFS mean d′**	**CFF->CFS *t*-value**	**Size cm^∧^2**
SOG	Inferior occipital gyrus and sulcus	0.22	3.39^*^	0.19	2.24^*^	23.96
LG	Lingual gyrus	0.04	0.39	0.13	2.19^*^	42.04
OP	Occipital pole	0.27	2.58^*^	0.22	3.33^*^	38.05
CTPS	Posterior transverse collateral sulcus	0.35	3.20^*^	0.25	2.90^*^	8.36
MLOS	Middle occipital and lunate sulci	0.22	2.21^*^	0.12	1.38	17.84
	**Full ROI name**	**VIS->CFS mean d′**	**VIS->CFS *t*-value**	**VIS->CFF mean d′**	**VIS->CFF *t*-value**	**Size cm^∧^2**
IOGS	Inferior occipital gyrus and sulcus	0.28	3.64^*^	0.50	4.06^*^	23.96
MOG	Middle occipital gyrus	0.23	3.06^*^	−0.03	−0.31	33.69
FG	Fusiform gyrus	0.28	3.82^*^	0.38	5.24^*^	27.08
LG	Lingual gyrus	0.25	3.06^*^	−0.06	−0.48	42.04
OP	Occipital pole	0.24	2.57^*^	0.12	1.32	38.05
CTPS	Posterior transverse collateral sulcus	0.50	3.84^*^	0.32	2.05^*^	8.36
MLOS	Middle occipital and lunate sulci	0.17	2.28^*^	0.32	2.47^*^	17.84
AOS	Anterior occipital sulcus	0.33	2.67^*^	−0.18	−1.79	13.24
LOTS	Lateral occipitotemporal sulcus	0.27	2.52^*^	0.49	3.47^*^	17.66

* *Indicates FDR-corrected p < 0.05*.

In the invisible to visible case, that is, training on trials from either invisible method and testing on only visible trials, we were unable to find any ROIs that could distinguish the stimulus categories reliably. When training on visible trials and testing on invisible trials, however, we found that several occipital and temporal ROIs could distinguish the categories (Figure 4; table of *t*-values and mean accuracies for ROIs found in Table 2). Training on visible trials and testing on either invisible presentation method yielded significant classification results in the inferior occipital gyrus and sulcus [VIS->CFF: *t*_(16)_ = 4.06, VIS->CFS: *t*_(16)_ = 3.64, both *p* < 0.05], fusiform gyrus [VIS->CFF: *t*_(16)_ = 5.29, VIS->CFS: *t*_(16)_ = 3.82, both *p* < 0.05], posterior transverse collateral sulcus [VIS->CFF: *t*_(16)_ = 2.05, VIS->CFS: *t*_(16)_ = 3.84, both *p* < 0.05], middle occipital and lunate sulci [VIS->CFF: *t*_(16)_ = 2.47, VIS->CFS: *t*_(16)_ = 1.90, both *p* < 0.05], and in the lateral occipitotemporal sulcus [VIS->CFF: *t*_(16)_ = 3.46, VIS->CFS: *t*_(16)_ = 2.52, both *p* < 0.05]. In four other regions, all in the occipital lobes, reliable visible to invisible categorical classification only occurred when the classifier was tested on CFS trials [middle occipital gyrus: VIS->CFF: *t*_(16)_ = −0.31, *p* > 0.05, VIS->CFS: *t*_(16)_ = 3.31, *p* < 0.05; lingual gyrus: VIS->CFF: *t*_(16)_ = −0.48, *p* > 0.05, VIS->CFS: *t*_(16)_ = 3.06, *p* < 0.05; occipital pole: VIS->CFF: *t*_(16)_ = 1.31, *p* > 0.05, VIS->CFS: *t*_(16)_ = 2.57, *p* < 0.05; anterior occipital sulcus: VIS->CFF: *t*_(16)_ = −1.79, *p* > 0.05, VIS->CFS: *t*_(16)_ = 2.67, *p* < 0.05]. For three of these regions, the classifier performed better with CFS test data than with CFF test data [paired *t*-test, middle occipital gyrus: *t*_(16)_ = 2.31, *p* < 0.05; lingual gyrus: *t*_(16)_ = 2.11, *p* < 0.05; anterior occipital sulcus: *t*_(16)_ = 3.63, *p* < 0.05].

In summary, cross-method category classification was possible exclusively in occipitotemporal cortex. No ROIs were capable of distinguishing visible category trials when classifier training occurred using either invisible method and testing was done using fully visible stimuli. Category information was recoverable when training and testing was done on data collected using different invisible presentation methods only within occipital cortex. Several occipitotemporal ROIs could distinguish face-related from tool-related activity when training a classifier on patterns of activity elicited by visible trials and testing the classifier using patterns elicited by either CFS or CFF trials; however, activity in the middle occipital gyrus, lingual gyrus, and anterior occipital sulcus could reliably predict category when classifier testing was done using CFS trials, and was also reliably more robust than when testing on CFF trials.

## Discussion

We presented two categories of objects (tools and faces) to subjects while scanning them using fMRI. Objects were rendered invisible using two methods, CFS and CFF, and were also presented during normal viewing. We used MVPA on the fMRI data to identify anatomically defined areas in which patterns of activation elicited by each presentation method allowed us to recover stimulus category information. Even though there were differences at the level of stimuli when presenting categories using CFS and CFF, there were no differences in the set of face or tool images made invisible using either method. Moreover, we did not classify brain activity by method, but rather by stimulus category. Thus, stimulus differences were orthogonal to our comparisons, and cannot account for our results. Here we show that distinct cortical regions carry information about visual categories when visibility is eliminated by each of these two methods. This result confirms previous findings that neural information processing can occur when stimulus awareness is obstructed by both CFS and CFF (Fang and He, 2005; Jiang et al., 2007; Sterzer et al., 2008), and shows for the first time, that these two different methods for rendering stimuli invisible can activate different, largely non-overlapping, cortical areas. This suggests that cortical representations at the level of categories vary with presentation method.

Our visible classification results identified a neocortical network of occipitotemporal, parietal, and lateral frontal regions that could reliably distinguish faces from tools when the objects were presented under normal viewing conditions and were clearly visible. In several of these regions, not only were the results significant, but they were also significantly stronger than during either invisible case. These results are unsurprising and broadly consistent with previous research. Lateral occipital and superior parietal cortex have both been shown to activate strongly to tools (Vingerhoets et al., 2009; Mahon et al., 2010) and inanimate object stimuli more generally (Chao et al., 1999). Our finding that visible face and tool stimuli can be reliably distinguished from each other in medial and lateral occipitotemporal cortical ROIs, as well as in the intraparietal sulcus, is consistent with these results. A large amount of evidence also suggests that faces robustly activate lateral fusiform regions in the temporal cortex, among other regions (Haxby et al., 2000). Our visible classification results in the middle frontal gyrus show the presence of categorical information outside of these regions, specifically in the middle frontal gyrus and in the superior part of the precentral sulcus.

The category classification results when subjects were not aware of the stimuli are more interesting. When stimuli were suppressed using CFS, category could be recovered exclusively from parts of the occipital lobes, but when CFF was used to keep stimuli from awareness, category information could be recovered from medial occipital, lateral temporal, and lateral frontal cortex. In addition, for both invisible presentation methods, at least one region was found that showed the presence of stronger categorical information than the other invisible presentation method. The only region that could reliably distinguish the presented categories across all three presentation methods was the lingual gyrus, on the medial surface of the occipital lobe. This area comprises part of the extra-striate visual cortex (V2-V4) and our finding is consistent with the role of these regions in lower-level processing of visual objects (Grill-Spector and Malach, 2004).

Our CFS results are inconsistent with previous findings that category-level information about stimuli suppressed with CFS is available within the ventral temporal cortex (Sterzer et al., 2008, 2009), and with other results linking the presence of tool-related categorical information within the posterior parietal cortex during CFS (Fang and He, 2005). We found that categorical information in the CFS condition was available in much earlier visual regions, all exclusively occipital. This specific result is consistent with a recent study that showed that information is restricted to occipital cortex under CFS and does not extend to either higher-level regions along the ventral and dorsal streams (Hesselmann and Malach, 2011). We found no evidence of categorical information being represented in the superior parietal cortex during CFS. Furthermore, the categorical signal in this region was significantly stronger during visible trials compared to CFS trials. One possibility is that our classifier relied on differences between the categories that were not strictly semantic or categorical, but based on lower-level visual features. However, we attempted to mitigate these kinds of effects by using training and test datasets that contained distinct sets of exemplars from the same category. We also used line drawing images that were not as detailed as those used in the studies mentioned above, which may explain the inconsistencies between our and their results.

The results of the CFF within-method classification were quite different from what was found with the CFS data, and contradict the hypothesis that categorical information about the stimuli was restricted to early visual areas. Information about CFF categories could be recovered both in the fusiform gyrus, a region in temporal cortex that has been consistently implicated in higher-level, categorical processing of visual objects, especially faces (Kanwisher et al., 1997), and in the superior part of the precentral sulcus of the frontal lobe, which has been implicated in attentional control over visual stimuli (Thompson et al., 2005). A previous fMRI study of CFF compared neural responses to the same stimuli when perceived to be flickering vs. fused, and found that frontal and parietal areas show greater activity to stimuli that appeared to be flickering (Carmel et al., 2006). Others have found that several regions in visual cortex can distinguish fused chromatic flicker from a matched non-flickering control, even when observers cannot (Jiang et al., 2007). In our experiment, we took a slightly different approach by presenting faces and tools that flickered in the same manner, and were seen as fused (and thus invisible) on nearly every trial. This allowed us to ask what areas carried information about the flickering stimuli, rather than the presence or absence of flicker *per se*. This approach has been more commonly used with CFS, where studies have found evidence of category information in ventral temporal and posterior parietal areas (Fang and He, 2005; Sterzer et al., 2008).

We found that several areas outside visual cortex could distinguish between the categories when they were visible, with one of these regions in frontal cortex capable of distinguishing between them during CFF. What stimulus information could be driving classification during each of these presentation methods? Several possibilities exist. The face and tool image sets were identical across visible and both invisible conditions. Moreover, the spatial extent and average luminance of the images was constant between categories, and the exemplars used for classifier training and testing were distinct. Our results are consistent with the possibility that classification is driven by category-level differences, although there remains the possibility of lower-level confounds because of stimulus-level differences. These could be shape-level category differences (e.g., tools were oblong and vertical, whereas faces were rounder, although also vertical) or semantic-level category differences. Shape-level category differences could cause differential activation in areas that process shape or in any region where clear visual topographic maps are known to exist, including in the occipital lobes, posterior parietal lobes, and in the frontal and supplementary eye fields (FEF/SEF) (Hagler and Sereno, 2006; Kastner et al., 2007; Wandell et al., 2007).

Cross-method category classification between the two invisible presentation methods was restricted to ROIs located in posterior occipital cortex, with no other ROIs showing above-chance classification regardless of which method was used as the training/test stimulus. This suggests that only in retinotopically organized areas encoding low-level visual features such as contour boundaries or overall image extent did the CFS and CFF presentations give rise to similar neural representations. Cross-method classification between visible and invisible presentation methods was possible in both occipital and ventral temporal cortex, but this effect only reached significance when the classifier was trained on visible trials and tested on invisible trials. When training on invisible trials and testing on visible trials there was no above-chance classification anywhere. This imbalance may arise because only the more robust stimulus signal available during visible trials is sufficient for training the pattern classifier. Even so, this result suggests that information about both the visual features and the category-relationship of the stimuli are represented in comparable ways between visual and invisible stimuli. Taken together, the cross-method classification analyses reveal an interesting distinction: The two invisible presentation methods only lead to shared representations in regions relatively early in the visual processing stream (lingual gyrus, middle occipital gyrus, middle and lunate occipital sulci), that code information about visual features. On the other hand, each invisible presentation method shares representations with visible presentations in both early visual and later areas that support more category-oriented encoding. This suggests that at least some degree of category information is attained within each invisible presentation method, but that there is a divergence in how category-level information is represented between the two methods after the shared early representation of visual features.

In conclusion, we show that the method used to render a given stimulus invisible has a significant effect on the way in which information about that stimulus can be recovered from neural activity within the human brain. The only region that allowed the recovery of information about object category using all three methods was the lingual gyrus, an area relatively early in the visual processing hierarchy. After this level there appears to be a divergence in bottom-up processing depending on the method used to attain invisibility. In the case of CFS, category information presumably propagates to the lateral occipital surface, and in the case of CFF, information presumably propagates into ventral temporal cortex and to the FEF.

Of the many “C areas” that permitted category decoding under conditions of conscious visibility, different subsets of “U areas” also permitted category decoding under various conditions of unconsciousness or invisibility (whether CFF→CFF, CFS→CFS, CFS→CFF or CFF→CFS). Removing these U areas from the former set of C areas leaves the following subset: anterior occipital sulcus, lateral occipitotemporal sulcus, intraparietal sulcus, and the middle frontal gyrus. It might be tempting to conclude that these “C-U” areas are necessary for conscious vision, and that U areas are not sufficient for conscious vision. However, we simply do not know what it is about neural processing in these areas that makes it possible to classify the category of visible objects, but not invisible objects. We also cannot rule out the possibility that some U areas play a necessary or even sufficient role for consciousness under visible conditions, arising from different forms of neural activity than those that allowed classification under conditions of invisibility in our experiment.

These results have implications for research into the limits of processing in the absence of awareness. They suggest that unconscious processing is not a single, unified phenomenon. Rather, where and how unconscious processing occurs is to a large extent dependent not only on the stimulus being presented, but also on the methodology used to present it. Caution is therefore needed before making strong claims about the nature of conscious or unconscious processing using only a single method for rendering stimuli invisible. Conversely, this should allow future researchers to tailor their stimuli for rendering stimuli invisible in a manner that attains the kinds of unconscious processing they wish to investigate.

### Conflict of interest statement

The authors declare that the research was conducted in the absence of any commercial or financial relationships that could be construed as a potential conflict of interest.
